# Meeting the Need for a Discussion of Unmet Medical Need

**DOI:** 10.3390/healthcare10081578

**Published:** 2022-08-19

**Authors:** Denis Horgan, Bettina Borisch, Bogi Eliasen, Peter Kapitein, Andrew V. Biankin, Stefan Gijssels, Michael Zaiac, Marie-Helene Fandel, Jonathan A. Lal, Marta Kozaric, Barbara Moss, Ruggero De Maria, Marius Geanta, Frédérique Nowak, Antoni Montserrat-Moliner, Olaf Riess

**Affiliations:** 1 European Alliance for Personalised Medicine, 1040 Brussels, Belgium; 2 Department of Molecular and Cellular Engineering, Jacob Institute of Biotechnology and Bioengineering, Faculty of Engineering and Technology, Sam Higginbottom University of Agriculture, Technology and Sciences, Prayagraj 211007, India; 3 Institute of Global Health, Faculty of Medicine, University of Geneva, 1202 Geneva, Switzerland; 4 Copenhagen Institute for Futures Studies, 1473 Copenhagen, Denmark; 5 Inspire2Life, 3991 CD Houten, The Netherlands; 6 Wolfson Wohl Cancer Research Centre, Institute of Cancer Sciences, University of Glasgow, Garscube Estate, Switchback Road, Bearsden, Glasgow G61 1QH, UK; 7 Patient Expert Centre (PEC), 1930 Zaventem, Belgium; 8 Novartis Pharma AG, 4056 Basel, Switzerland; 9 Sanofi, 1831 Diegem, Belgium; 10 Department of Genetics and Cell Biology, Institute for Public Health Genomics, GROW School of Oncology and Developmental Biology, Faculty of Health, Medicine and Life Sciences, Maastricht University, 6211 LK Maastricht, The Netherlands; 11 Bowel Cancer UK, London SE11 5DP, UK; 12 Alleanza Contro il Cancro, 00161 Rome, Italy; 13 Faculty of Medicine, University of Medicine and Pharmacy ”Carol Davila”, 050474 Bucharest, Romania; 14 Coordination du Plan France Médecine Génomique 2025, Inserm, 75013 Paris, France; 15 European Commission, 1471 Luxembourg, Luxembourg; 16 Institute of Medical Genetics and Applied Genomics, University of Tübingen, 72076 Tübingen, Germany

**Keywords:** unmet medical need, personalised medicine, innovation, policy framework, regulation

## Abstract

As Europe and the world continue to battle against COVID, the customary complacency of society over future threats is clearly on display. Just 30 months ago, such a massive disruption to global lives, livelihoods and quality of life seemed unimaginable. Some remedial European Union action is now emerging, and more is proposed, including in relation to tackling “unmet medical need” (UMN). This initiative—directing attention to the future of treating disease and contemplating incentives to stimulate research and development—is welcome in principle. But the current approach being considered by EU officials merits further discussion, because it may prove counter-productive, impeding rather than promoting innovation. This paper aims to feed into these ongoing policy discussions, and rather than presenting research in the classical sense, it discusses the key elements from a multistakeholder perspective. Its central concern is over the risk that the envisaged support will fail to generate valuable new treatments if the legislation is phrased in a rigidly linear manner that does not reflect the serpentine realities of the innovation process, or if the definition placed on unmet medical need is too restrictive. It cautions that such an approach presumes that “unmet need” can be precisely and comprehensively defined in advance on the basis of the past. It cautions that such an approach can reinforce the comfortable delusion that the future is totally predictable—the delusion that left the world as easy prey to COVID. Instead, the paper urges reflection on how the legislation that will shortly enter the pipeline can be phrased so as to allow for the flourishing of a culture capable of rapid adaptation to the unexpected.

## 1. Introduction

The European Commission announced in 2020 its intention for a major review of the EU’s pharmaceutical legislation, including possible shifts in its approach to tackling unmet medical need (UMN). The preparations for drafting proposals have been ongoing since then [1]), notably featuring a public consultation from 30 March 2021–27 April 2021 and a further consultation from 28 September 2021–21 December 2021. In the summer of 2022, the Commission plan was to propose a draft legal text in the fourth quarter of 2022. This may seem cumbersome, but it is consistent with the complex decision-making process in EU legislation. A Commission proposal based on extensive consultations is presented to the European Parliament and to the Council—the body composed of member state governments—who each have to reach a view on the proposal and then have to reach an agreement between themselves on any divergences in their respective positions before the legislation becomes final. The process, typically lasting five years (but often much more) from concept to finalisation, involves numerous further discussions to refine the proposals and bridge divergences at different levels and in different forums, involving officials, diplomats, politicians and stakeholders. In the case of health-related legislation, the stakeholders include patients, healthcare professionals, healthcare providers, technology developers, health technology assessment bodies, insurance organisations and other organisations that pay for healthcare and civil societies and other organisations representing the healthcare community—including European Alliance for Personalised Medicine (EAPM).

Coincidentally, this legislative review is taking place against the background of the COVID outbreak, lending particular significance to the topic of UMN—with the surprise element in the speed, scope and scale of that pandemic still resonating across Europe and around the world. The recent declaration of monkeypox as a major health threat only adds to the importance of the debate [2,3].

The record of health policymakers in tackling unexpected health events had not been glorious over recent years, and the COVID experience did nothing to add to the lustre. A few lone voices had been warning of the notional risk of a major pandemic for years, but as earlier threats such as Severe Acute Respiratory Syndrome (SARS) [4] and the Swine flu pandemic (H1N1 flu) [5] melted away, anxiety gave way to indifference and played into the natural human behaviour of ignoring warning signs, hoping for the best and carrying on as normal. The consequences of that miscalculation are now obvious, demonstrating that, in matters of health (as in matters of geopolitics or the environment), the unexpected cannot be safely discounted. Now Europe faces another decision point in the health arena.

One of the explicit objectives of the European Union review is—as the European Commission has expressed it—to “foster innovation, including in areas of UMN”. The ambition is laudable, because pharmaceutical innovation has generated huge improvements in public health over the course of a generation, and Europe has good chances of bringing in many further advances if the environment is conducive [6,7,8,9] However, UMN is subject to many different perceptions and interpretations by different stakeholders. It has a different meaning for regulators, health technology assessment experts, payers, the pharmaceutical industry and also for patients, all of whom are debating how to formulate a proper definition or a set of principles for “unmet medical needs”. This argues for a step back from heedless legislation on how to support UMN [10]. 

At first sight, it may seem churlish, even perverse, to offer any criticism of a policy geared to meeting unmet need. There are already so many clearly identified unmet needs that are urgent, even desperate; who could argue against it? [11,12,13,14,15]. The Commission has already listed some of the obvious targets—notably, rare diseases and antimicrobial infections [16,17,18]. Few would disagree with the list, and many would add many more pathologies. The difficulty emerges only when the limitless possibilities for fostering innovation run into the brick wall of the inevitable limitation of resources for support. At present, EU support for such research, focused on rare diseases and paediatric diseases, offers a menu of incentives (fee-free scientific advice, exemption from other fees, extended protection against copying…), all of which have a direct or indirect impact on the financing of healthcare and the perennial problem of demand exceeding supply [19,20].

But the problem does not lie in the concept of support for innovation. The problem arises only if the decisions on the allocation of that inevitably limited support were to be based on questionable logic and inappropriate criteria. The purpose of this paper is not to prescribe how those decisions should be made or exactly which criteria should apply. It aims merely to provide informed guidance to policymakers for the upcoming discussions. It does not attempt to provide definitive answers but can serve to offer caution over potential pitfalls on the basis of wide multi-stakeholder experience. If the focus on “unmet need” is rigidly prescriptive and becomes a condition for qualifying for support (and there are unofficial indications that current thinking tends that way within parts of the Commission), serious reflection is needed on what criteria might be deployed in making those judgment calls on who gets what out of mechanisms designed to “foster innovation”. Is it, in fact, possible to define watertight criteria? If such precise criteria are to be generated, would they risk being counterproductive by excluding innovation that might, over the course of time, prove to be capable of meeting still greater—but as of yet unrecognised—unmet needs? [21,22,23,24,25].

## 2. Destinations and Routes

If it is considered that innovation should be fostered—and there is little dissent from that—the question arises as to how this could best be done. The revision of the legislation is about all medicines—orphans and non-orphans—and the definition of UMN will play a key role in determining the level of regulatory incentives a product gets in terms of the protection of regulatory data and market protection for non-orphans, as well as market exclusivity for orphans [26,27]. The current legislation, providing, in most cases, for a “one size fits all” approach, is somewhat blunt, but it has the merits of offering some predictability. Now, the mood-music from the Commission, as it mulls its proposals due at the year’s end, is shifting to a modulated approach more explicitly linked to UMN and based on defined criteria. According to the latest suggestions circulating, the health officials in the Commission are considering tightening orphan designation with respect to the prevalence threshold employed and introducing a time-limit to its validity [28]. They are also contemplating imposing new time limits on the market exclusivity orphans enjoy for most products (except those considered to be addressing high unmet needs—and even those would have to demonstrate lack of return on investment and be marketed promptly across the EU). Furthermore, the Commission are considering reducing the baseline for regulatory data protection from 8 to possibly 6 years. Products would receive additional years of regulatory data protection depending on whether the product addresses an unmet medical need, and/or is launched in all 27 Member States. It is unclear yet whether other parts of the Commission are willing to endorse it considering the potential impact on research and innovation in the EU—illustrating clearly just how challenging it is to find a single simple algorithm. 

This leads to the fundamental question as to whether this will steer research and development (R&D) towards products that benefit patients across the EU. Are there other benefits that could be envisaged, over and above regulatory support schemes at the pre-authorisation phase and the additional regulatory protection periods at the post-authorisation phase? How feasible—and beneficial—would it be to establish a list of UMNs of specific therapeutic areas or conditions to steer the development of medicines in these areas? How widely should the criteria range? Should the burden, or costs, of a disease on the healthcare system be taken into account? If so, who should compile this list and keep it updated? How attractive might the option—currently being mooted—of transferable vouchers as a reward for developing orphan medicines deemed of high UMN be?

The ultimate touchstone in this exercise is whether the revision will be helpful to drive forward innovation and ensure access. For the European Alliance for Personalised Medicine (EAPM) and for the European Commission as well, personalised medicine is considered to be the right prevention and treatment for the right patient at the right time [6,29]. How compatible will a restrictive system of incentives be with that objective?

## 3. Devils and Details

One of the key questions in this context must be how to define UMN. On this, there is a lack of alignment, and as a result, tensions exist between stakeholders and decision makers across a wide range of contexts where the concept is applied [15,30]. Every patient with unsatisfactory treatment options is naturally going to consider this to be a case of UMN. Narrowing the principle of unmet need—for instance, to suggesting that, where a treatment already exists, there is no more medical need—does not serve that patient’s interests [31,32]. It is hardly adequate to consider a need as met when treatments already exist and may prolong the lives of oncology patients but not for long enough or with treatments of which the side effects or burden are not acceptable. Each patient is different and may need a different treatment. Another question is how sensible it is to discuss UMN in isolation from the drivers of innovation that condition the possibility of meeting a need. Innovations result from public and private investments and are governed by the speed, quality and ingenuity of R&D. It is not possible to separate the aspirations from the reality, and there is an evident danger in trying to disconnect the discussion from the process of scientific evolution or pre-empt that essential aspect of seeking to meet needs. A rigid framework would disregard the evolution of science. A positive list of UMNs would lose contact with new discoveries coming out of evolving science. Yet another question is how possible it is to reliably predict the future. This is an essential consideration in any creation of definitions because of the very nature of innovation—which is essentially unpredictable. Since it takes, on average, more than a decade to develop a new medicine [33], along a process that is itself highly unpredictable, with no guarantees that the identified need will be met by any eventual product and with the possibility that another previously unidentified need may indeed be met by the outcome, rigid definitions have limitations. As an example, when research started on mRNA technology, few would have imagined it would play a central role in fighting a pandemic [34]. The unexpected emergence of COVID is only one example of the unpredicted and unpredictable. Another is monkeypox [35], and the experiences with Zika [36] and Ebola [37] are also still fresh in the memory. Thus, UMN would have to be defined continuously and in rather short timeframes.

The only firm prediction that is logically possible is that the unpredicted will continue to emerge—implying that there is peril in discouraging or even closing off research assistance and resources because of an essentially arbitrary decision over what is an unmet need and what is not. In fact, decisions over resource allocation might be construed as more of a political question, since, in the case of many diseases, where there has been an evident need to act, the need is often not met until there is a political need to do so—as with COVID when it reached the EU and North America. Deciding on what is an “unmet need” can be cynically described as a value question linked to incremental innovation, where resources need to be rationally allocated. Excessively prescriptive limits to the award of incentives could prove to be at best ill-targeted and at worst downright counterproductive. If the Commission were to follow through on its hypothesis of creating a list of therapeutic areas recognised as representing an unmet medical need in the EU, the question would arise as to how such a list would help in averting health threats that are unknown today. There are also obvious questions over how satisfactorily and agilely patients’ perspectives, preferences and insights would be integrated into such a list. Effective discrimination among patients could also emerge as a risk where meaningful regulatory incentives would apply only to therapeutic areas featured on a list. Potentially, there is also the risk that only mainstream projects will be prioritised, neglecting small innovations, for instance, on compound structure modelling or AI-based prediction of treatment efficacy, which could potentially play a major role in the future. There is another aspect to the debate linked to the future of Europe’s own innovative capacity. It is already clear that the EU is losing ground internationally in innovation, with only 20% of innovative medicines coming from the EU compared to 50% from the US—a stark reversal of the situation 25 years ago, when Europe had a clear lead [38]. Meanwhile, competition from Asia and, in particular, China is growing [39]. If a more restrictive regulatory incentives system were to be put in place in Europe, it could make it even more challenging, and possibly less rewarding, for pharmaceutical developers to file their product approval applications in the EU. The threat to innovative medicines addressing all types of UMNs is obvious: they may not continue to be filed in the EU. It might be prudent for the Commission to stress-test its concept before making its proposals. Ultimately, the most appropriate definition of UMN would be: “UMN refers to any medical condition that is not adequately treated or diagnosed by authorised interventions” [30]. There are many such circumstances and conditions, and the solution depends on innovation. Addressing UMNs and creating innovations correlate closely, as evidenced by recent advances in patient care—such as the HPV vaccine, which reduced the risk of cervical cancer by almost 90%, or treatments that transform the response to melanoma, for which only 5% of patients would survive five years after diagnosis, while today there are 50% survivors. The innovative course of medicine is the consequence of addressing UMNs in the first place [40].

What the EU needs to accelerate innovation is not a limit to the definitions of unmet need but a broad and flexible definition based on principles defined by all stakeholders working together in stronger public–private partnerships in a transparent and predictable regulatory framework. To learn from the past is prudent, but it is folly to assume that knowledge of the past confers omniscience about the future.

## 4. Discussion

There are complications to the debate about UMNs that risk confusing what is by its nature a complex topic. It is taking place in an atmosphere that has grown heady with eloquently expressed and often understandable demands for increased rights that are sometimes controversial, sometimes less than realistic and sometimes downright conflictive or contradictory. Specifically in the area of UMNs, the aspirations include rights for one group of patients or another or rights to the access of treatment. These play into and are energised by wider aspirations on healthcare-related issues as diverse as rights to data protection, rights to share data, rights to patient empowerment or even the right to have a return on investment [41]. Such rights are easily proclaimed, but it has to be borne in mind that there are asks which might make sense on paper and sound good as slogans but cannot be so easily realised. Proclaiming a right is very different from implementing the mechanisms needed to turn aspirations into reality. Crudely put, the “rights” of any patients are strictly and objectively limited by factors that no amount of protestation can influence: the level of member state competence, the state of scientific understanding of particular diseases and the characteristics of individual patients and the stage of their disease; even the hot-button topics of the access to and the affordability of care are limited by time, place and resources. The right to a return on investment depends on innumerable factors beyond the direct control of investors—everything from product quality to research efficiency, market conditions, earthquakes, and fires. The ease with which “rights” can be announced these days carries, at worst, the risk of breeding a certain disdain for reality—even a facile presumption that earnestness of appeal somehow equates to the availability of a solution. The presumption is often associated with the suspicion—or even the conviction—that established institutions are somehow allied against the interests of the people in deliberately frustrating the claimed rights.

This unhelpful phenomenon should be avoided in the current discussions on reviewing the EU’s pharmaceutical legislation [1] and particularly its support for innovation [42]. The European Union has repeatedly acknowledged that its manifest unreadiness to face the COVID pandemic at its outset now demands a deliberate upgrade of readiness to face an unpredictable future. This determination lies behind the decision to create the European Health Emergency Preparedness and Response Authority (HERA), which in its very title reflects the recognition of unlimited potential risk. Europe did manage, on this occasion, by heroic efforts, to deliver partial solutions to the challenges of COVID, and that has provided the healthcare community and decision makers some time for reshuffling and regrouping forces. However, on the EU’s own admission, this has been achieved only at the cost of hundreds of thousands of lives and tens of millions of cases of serious disease (to say nothing of some two years of severe economic and social disruption and huge personal suffering). The EU’s efforts at home have so far been echoed only to a limited extent in countries beyond its borders. So, it is only a very mitigated success.

As a matter of policy, the EU is now committed to trying to get ahead of the curve on health threats, and it has buckled the concept firmly onto its bid to establish a European Health Union. The work of the HERA now consists, in large part, of working on future readiness with the innovative pharmaceutical sector—essentially, the pharmaceutical companies that came up, in extremis, with vaccines and therapeutics to counter COVID [6,7]. The development of many of those products was assisted by EU funding and regulatory support that amounted to a calculated gamble with the unknown. The vaccine and therapeutic strategies adopted in this emergency provided support in terms of hard cash for defined objectives—but not for how the objectives were to be achieved. Those vital aspects were left to the companies that were conducting the research and development. By good fortune, most of that work paid off in terms of valuable products, but at the time, the support was given as essentially an act of faith in innovation. How bizarrely inconsistent would it be for the same EU to now opt for a restrictive approach to supporting the innovation on which much of the success of its preparedness necessarily depends? With respect to the realities of innovation, the EAPM urges the EU to be supportive of innovation—and, of course, of innovation that tackles current unmet needs—but to avoid limiting support for innovation that fails to meet criteria that are set today but disregard tomorrow.

The history of EU healthcare legislation is marred by some striking examples of prematurely adopted rules that subsequently proved to be ill-thought-through and have led to subsequent confusion, complications and the need for correction. Ill-considered and over-hasty rules enacted back in 2000 that aimed at encouraging clinical trials were almost immediately revealed to be counter-productive, impairing Europe’s CT performance, and required comprehensive replacement in 2016 with a new regulation (which in itself was so imperfectly drafted that it finally came into effect only at the end of January 2022). The General Data Protection Regulation (GDPR), also enacted in 2016 to protect personal data, was also so imperfectly drafted that it unintentionally handicapped much of the secondary use of health data in Europe and left researchers battling against a deeply fragmented regulatory landscape with divergent rules implemented across the Member States [43,44]. Such salutary examples should serve as a warning against over-hasty or over-simplified attempts to approach UMN as a Gordian knot that can best be unloosed by a single sharp blow [42].

## 5. Conclusions

It may be considered disappointing that this paper offers no specific solution to the problem it identifies, but it is in the nature of a panorama of some of the divisive issues where UMN overlaps with innovation that the conclusion should do no more than urge further discussion involving all stakeholders. It does not pretend or presume to provide a one-size-fits-all approach or a rigid definition, because that may distort the path that should now be travelled by all stakeholders to get to the causal factors that prevent or slow down a given disease and that condition the emergence of innovative medical responses. Whatever emerges from the legislative process needs to be accepted by a wide range of diverse European views and, notably, patients. So, the only direct recommendation here is that the forthcoming legislative debates should be conducted with an openness to examining the many distinct points of view on UMN in an honest attempt to identify answers that do not carry the risk of unforced errors that would impede innovation. The process of policy formation should be slow, with dialogue, as needed, among stakeholders with legitimate views that deserve to be heard and considered. What is vital is to avoid policymakers blindfolding themselves to the unpredictability of diseases and ignoring the underlying realities of the innovation process that are clearly understood by the scientific and research community and by the broad range of health stakeholders.

## Data Availability

Not applicable.
